# Spatial Assessment of Anthropogenic Impact on Trace Metal Accumulation in Farmland Soils from a Rapid Industrializing Region, East China

**DOI:** 10.3390/ijerph15092052

**Published:** 2018-09-19

**Authors:** Wei Jiao, Yong Niu, Yuan Niu, Hengyu Hu, Ruiping Li

**Affiliations:** 1 Shandong Provincial Key Laboratory of Water and Soil Conservation and Environmental Protection, College of Resources and Environment, Linyi University, Linyi 276000, China; hhyu01@163.com; 2 Institute of Lake Environment, Chinese Research Academy of Environmental Sciences, Beijing 100012, China; ny0626@outlook.com (Y.N.); niuyuan@craes.org.cn (Y.N.); 3 School of Geography and Tourism, Qufu Normal University, Qufu 273100, China

**Keywords:** trace metals, anthropogenic impact, spatial distribution, enrichment factor, principal component analysis

## Abstract

A better understanding of anthropogenic trace metal accumulation in farmland soils is crucial for local food safety and public health, especially for a rapidly industrializing region. In this study, soil samples at two depths were collected from a typical county in East China and analyzed for total concentrations of Fe, Al, Pb, Cd, Cu, Zn, Cr, and Ni. Results showed that trace metals like Pb, Cd, Cu, Zn, Cr, and Ni have accumulated in the regional farmlands, with average topsoil concentrations 1.62–1.77 times higher than their background concentrations in subsoil. However, they were still much lower than the limits of the Chinese Environmental Quality Standard for Soils. By the proper calculation of enrichment factor (EF), it was found that the accumulations of trace metals in the topsoil have been impacted by anthropogenic activities, which could contribute up to 40.83% of total metal concentration. Two principal components were extracted according to the results of principal component analysis (PCA) for EF values, which indicated two important anthropogenic trace metal sources. With the help of spatial distribution maps based on geographical information system (GIS), the anthropogenic sources of Pb, Cr, and Ni were determined to be mostly associated with atmospheric deposition from the central urban area. However, Cd, Cu, and Zn were further confirmed to originate from different agricultural sources. The anthropogenic Cu and Zn inputs were mostly related to pig manure application in the rural northern and southeastern areas, while extensive fertilizer application was identified as the major contributor to anthropogenic Cd accumulation in this region. Overall, the integrated application of EF, PCA, and GIS mapping is an effective approach to achieve the spatial assessment of anthropogenic impact on trace metal accumulation in regional soils.

## 1. Introduction

The accumulation of trace metals in farmland soils is of increasing concern in China due to its potential threat to food safety and public health [1,2]. Under natural conditions, the trace metals in soil are mostly related with parent materials, which thus have limited mobility [3]. However, those originating from anthropogenic sources are believed to be more active and easier to transfer from soil to human through the food chain [4]. Therefore, serving the effective reduction of environmental and human health risk, a key question we must make clear is how the anthropogenic activities have actually increased trace metal concentrations in the farmland soils.

Although undesired, the trace metal concentrations in farmland soils would increase gradually due to continuous anthropogenic inputs [5]. Such anthropogenic inputs usually include fertilization, manure application, sewage irrigation, and atmospheric deposition, among others [6,7,8]. It was estimated that animal manure application accounted for approximately 55%, 69%, and 51% of the total Cd, Cu, and Zn inputs to farmland soils in China, respectively, while atmospheric deposition was responsible for 43–85% of the total Pb, Cr, and Ni inputs [9]. In general, these data provide very valuable information for soil conservation at a national scale. Nevertheless, there will be uncertainty in terms of a small or specific region. The reason for such uncertainty is rooted in the fact that trace metal sources and their impact may vary greatly at different spatial scales [10]. To date, numerous methods including enrichment factor (EF), principal component analysis (PCA), and spatial distribution analysis based on geographical information system (GIS) have been well developed for regional soil monitoring purposes, which can help to identify and apportion the trace metals from anthropogenic sources [11,12,13].

Tancheng County is an important grain production base of Shandong Province in Eastern China. This county is rich in land resources and agriculture developed over a long time. Over the past 10 years, it has begun to experience a rapid transition from an agricultural-based economy to an industrial-based economy. The rapid industrialization may put strong pressure on the regional soil environment. Recently, the elevated trace metal levels in soil had been reported in some regions of Shandong Province, which generally indicated a close association with local anthropogenic activities [14,15]. However, there are very limited studies conducted in the traditional agricultural regions experiencing a rapid transition to an industrial-based economy. A better understanding of anthropogenic trace metal accumulation in these regions is crucial for local sustainable agricultural development. Rapid industrialization has occurred in Tancheng County, but its impact on anthropogenic trace metal accumulation in the farmland soils is still unclear.

In this paper, soil samples at two depths were collected from the Tancheng County and analyzed for Fe, Al, and trace metals Pb, Cd, Cu, Zn, Cr, and Ni. Three methods including EF, PCA, and GIS mapping were integrated to achieve the spatial assessment of anthropogenic impact on trace metal accumulation in soil. The specific objectives with this study were: (1) to discriminate between natural and anthropogenic trace metals in the farmland soils of this rapidly industrializing region; and (2) to identify the major anthropogenic sources and their spatial impacts.

## 2. Materials and Methods 

### 2.1. Study Area Description

**Figure 1:**
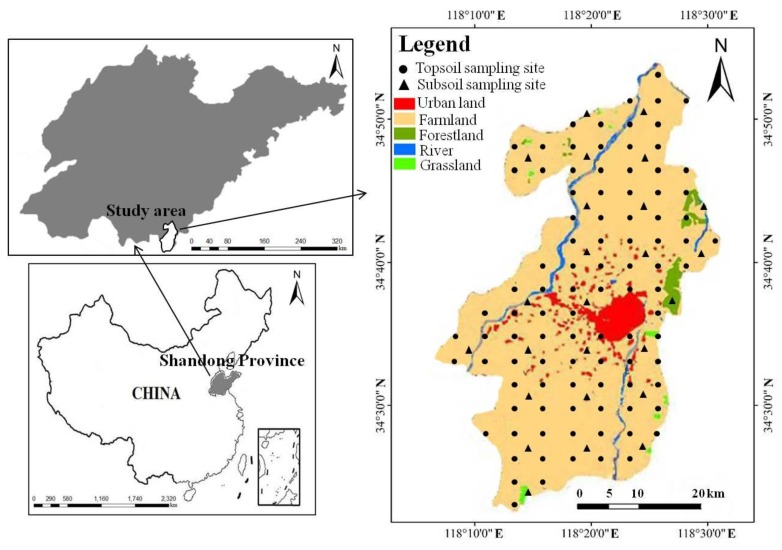
Location of the study area showing land uses and sampling sites.

Tancheng County is located in the south part of Shandong Province, East China, which covers 1195 km^2^ and has a total population of 0.97 million (Figure 1). This region is characterized by a typical temperate continental climate with the average annual temperature of 13.90 °C. The average annual rainfall is 919 mm, most of which occurs between June and September. Due to a huge alluvial plain in the central part, this region is generally flat and has an average elevation of 38 m. The fluvo-aquic soil, developed from river alluvial sediments, is the main soil type in this region. As an important grain production base of Shandong Province, Tancheng County has a long history of wheat and maize cultivation. All the irrigation water comes from natural rainfall, surface water, and groundwater. With the aim of regional economic structure adjustment, a rapid transition from an agricultural-based economy to an industrial-based economy has occurred in the last decade. Currently, it has established a preliminary industrial system containing chemistry, metal manufacture, paper production, and so on, most of which are located in the central urban area (see the Tancheng Government webpage: http://www.tancheng.gov.cn).

### 2.2. Soil Samples Collection

Based on grid cells, a total of 122 soil samples including 97 topsoil and 25 subsoil samples were collected from the regional farmlands in April 2017. The topsoil samples were collected from 0–20 cm in depth, with a density of 25 km^2^. At each sampling site, four to six subsamples near the cell center were taken and fully mixed to obtain a composite sample. The subsoil samples were collected at between 100 and 150 cm depth with a density of approximately 100 km^2^, which represent the natural background levels of the surrounding four topsoil samples. Geographic coordinates of all the sampling sites were recorded by a global position system (60CSX, Garmin, Olathe, KS, USA) (Figure 1).

### 2.3. Trace and Major Metals Determination

After transportation to the laboratory, all soil samples were air-dried, ground, and passed through a 0.149 mm nylon sieve. For analysis of the total concentrations of Pb, Cd, Cu, Zn, Cr, Ni, Fe, and Al, the dry samples were digested with an acid mixture of HNO_3_-HF-HClO_4_ (5:2:3, *v*/*v*/*v*) in Teflon tubes at 160 °C for 6 h and they were determined by inductively coupled plasma-atomic emission spectroscopy (ICP-AES; SPECTRO ARCOS EOP, SPECTRO Analytical Instruments GmbH, Kleve, Germany). The analytical data quality was assessed by simultaneously using the certified reference material GBW-07402. Comparisons between the measured and the certified values of the reference materials showed good agreement, with overall recoveries ranging from 97.12% to 104.78%. Furthermore, a sequential extraction procedure proposed by the European Community Bureau of Reference (BCR) was employed to determine the concentrations of Fe and Al associated to different chemical fractions in the 25 subsoil samples. The BCR procedure is described in detail elsewhere, which separates a metal into four operationally defined chemical fractions of acid-soluble, reducible, oxidizable, and residual [16]. By comparing the cumulative concentration of four chemical fractions with the concentration of the bulk sample, the overall rates ranged from 98.74% to 107.15%.

### 2.4. Enrichment Factor Calculation

The concept of “enrichment factor” (EF) was proposed in the 1970s to discriminate between natural and anthropogenic trace metals [17]. As a normalized index, it was originally applied to atmosphere and gradually extended to continental environments, such as soils or sediments [18,19]. The EF is defined as the ratio of a considered metal concentration to a reference metal concentration in a given sample, divided by the same ratio of their baseline concentrations.

(1) $$\mathrm{EF}\;=\frac{{\;(X/Y)}_{\mathrm{sample}}}{{\;(X/Y)}_{\mathrm{baseline}}\;}\;$$

where X is the total concentration of the considered metal and Y is the total concentration of the reference metal. Previous studies have suggested that the local soil background values can provide a more meaningful basis for calculating EF than that of the average crust values [20,21]. Therefore, concentrations of the considered and reference metals in the subsoil were adopted as the baseline values in this study. With the EF value for each trace metal, its anthropogenic contribution can be obtained as follows:

(2) $${\%X}_{\mathrm{anthropogenic}}\;=\;\frac{X_{\mathrm{sample}}{\;-\;Y}_{\mathrm{sample}}{\;\times \;(X/Y)}_{\mathrm{baseline}}}{X_{\mathrm{sample}}}\;\times \;100=\;\frac{\mathrm{EF}\;-\;1}{\mathrm{EF}}\;\times \;100\;$$

### 2.5. Statistical and Spatial Analysis

In order to select the best reference metal for EF calculation, the Spearman correlation analysis was conducted to evaluate the relationships between Fe, Al, and trace metals in the subsoil. Based on EF values, the PCA method was performed to identify the major anthropogenic sources for trace metals in the topsoil. According to the results of 97 topsoil sites, a spatial distribution map was established with ArcGIS (Version 10.0, ESRI Inc., Redlands, CA, USA) using ordinary kriging interpolation, which can help to assess the anthropogenic impact on trace metal accumulation in the whole region.

## 3. Results

### 3.1. Total Concentrations of the Trace Metals

A descriptive statistical summary of total Pb, Cd, Cu, Zn, Cr, and Ni concentrations in the topsoil and subsoil samples was presented in Table 1. In general, these trace metals have accumulated in the farmland soils of this rapidly industrializing region. The increases in Pb, Cd, Cu, Zn, Cr, and Ni were 77.19%, 72.73%, 62.03%, 70.43%, 68.34%, and 62.92%, respectively, when comparing their average concentrations in the topsoil with their concentrations in the subsoil. Among these metals, Zn exhibited the largest concentration both in the topsoil and subsoil. In addition, it was found that Zn had a larger variation coefficient than other trace metals in the topsoil, which was different from the case in the subsoil.

### 3.2. Enrichment Factors of the Trace Metals

#### 3.2.1. Selection of Reference Metal

Before EF calculation, it is essential to assess and select the best reference metal. Both Fe and Al are the most common reference metals when calculating EF. In this study, we selected Al as the reference metal according to a combined analysis of Spearman correlation and BCR chemical fractionation (Table 2). By comparison, it was found that Al exhibited a stronger positive correlation with those trace metals than Fe in the subsoil. Moreover, appropriately 84.13% of Al was observed in the residual fraction, which was far more than the residual Fe fraction and can therefore confirm its natural origin and conservative character.

#### 3.2.2. Calculation of Enrichment Factor

**Figure 2:**
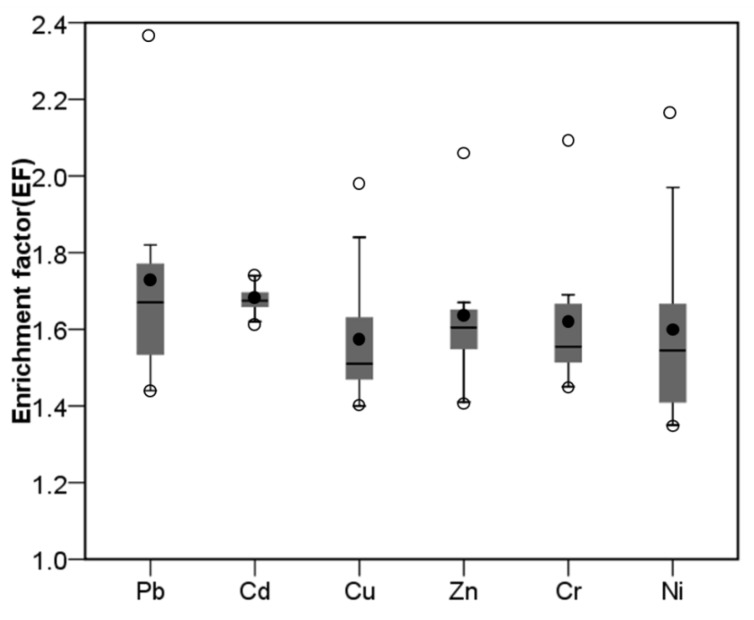
Enrichment factors of trace metals in the topsoil. Box plot (dark grey) extends from the 25th to 75th percentiles, covering the median (line) and the mean (black point) values; Circles at the top and bottom of box plot represent the maximum and minimum values; Whiskers represent the 5th and 95th percentiles.

By selecting Al as the reference metal, the EF values of Pb, Cd, Cu, Zn, Cr, and Ni in the topsoil were calculated relative to their background concentrations in the subsoil. As shown in Figure 2, the EF values varied among different trace metals, but they were all greater than 1.00. Because Pb had the highest average EF value (1.72), it was more enriched than other trace metals in the topsoil. The enrichment level of Cu was the smallest, which had an average EF value of 1.58. In general, the enrichment levels of these trace metals decreased in an order of Pb, Cd, Zn, Cr, Ni, and Cu.

#### 3.2.3. Estimation of Anthropogenic Contribution

With EF value, the anthropogenic contribution value was further estimated for each trace metal according to Equation (2). For Pb, its anthropogenic contribution values ranged from 30.37% to 57.60%, with an average value of 40.83%. Cd ranged from 38.09% to 42.49%, with an average value of 40.38%. Cu ranged from 28.56% to 49.38%, with an average value of 36.02%. Zn ranged from 29.06% to 51.56%, with an average value of 38.56%. Cr ranged from 31.08% to 52.11%, with an average value of 37.72%. Ni ranged from 25.86% to 54.02%, with an average value of 36.29%. For all the trace metals, the average contribution values of anthropogenic sources did not exceed their natural contributions.

### 3.3. Principal Component Analysis

The PCA method was performed to identify the major anthropogenic sources of trace metals in the topsoil. The input data were the EF values, which can provide more reliable information regarding anthropogenic impact than the total concentrations. The Kaiser-Meyer-Olkin (KMO) and Bartlett’s test results were 0.712 and *p* < 0.001, respectively, suggesting that PCA is effective in reducing the data dimensionality. As presented in Table 3, two principal components (PC1 and PC2) with eigenvalues greater than 1.000 were extracted, which explained 92.584% of the total data variance. As a result, all the trace metals can be well expressed by these two components. The first principal component PC1 explained 53.182% of the total data variance and was heavily loaded by Pb, Cr, and Ni. However, the second principal component PC2 was mainly dominated by Cd, Cu, and Zn, which accounted for 39.402% of the total data variance.

### 3.4. Spatial Distribution Maps

**Figure 3:**
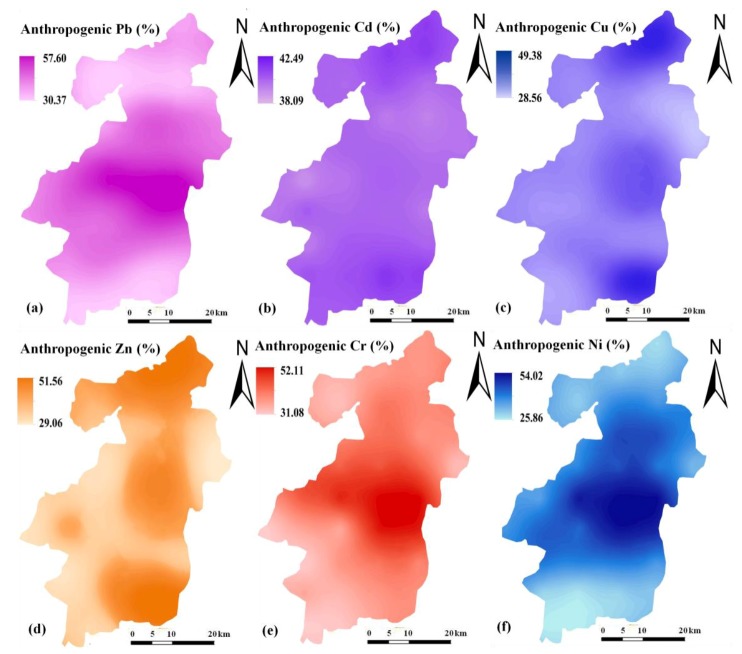
Spatial distribution patterns of anthropogenic Pb (**a**), Cd (**b**), Cu (**c**), Zn (**d**), Cr (**e**), and Ni (**f**) contributions in the topsoil.

Ordinary kriging method was used to map the spatial pattern of anthropogenic contribution to trace metal accumulation in the topsoil. Before this, a semivariogram analysis was conducted to select the most appropriate interpolation model. As shown in Table S1, different semivariogram models were selected according to trace metals, which generally satisfied the requirements of ordinary kriging interpolation because all the mean standardized prediction error (MSE) and root-mean-square standardized prediction error (RMSSE) values approached 0 and 1, respectively. The spatial distribution maps of anthropogenic contribution values are displayed in Figure 3. With regard to Pb, Cr, and Ni, they showed a similar spatial pattern, with high anthropogenic contribution values mainly distributed in the central urban area. However, such a distribution pattern was generally different from those of Cd, Cu, and Zn, which had higher anthropogenic contribution values in the rural northern and southeastern areas. By comparison, both Cu and Zn were observed to exhibit a larger spatial variability of anthropogenic contribution than that of Cd in the topsoil.

**Figure 4:**
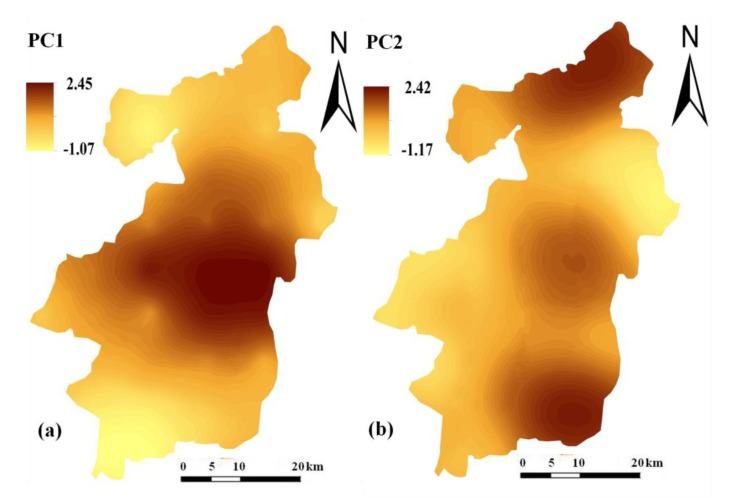
Spatial distribution patterns of PC1 (**a**) and PC2 (**b**) in the topsoil.

The spatial distribution patterns of the first two principal components were also displayed in Figure 4. It was found that the spatial distribution of PC1 was generally characterized by high values in the central urban area, which coincided with the spatial patterns of anthropogenic contributions to Pb, Cr, and Ni accumulation in the topsoil. However, higher scores of PC2 were mainly located in the northern and southeastern areas, where anthropogenic activities had an important impact on Cd, Cu, and Zn accumulation in the topsoil.

## 4. Discussion

### 4.1. Assessment of the Anthropogenic Trace Metal Accumulation in Regional Soils

Previous studies have indicated that anthropogenic activities are the major cause for excessive trace metal accumulation in soils, even on a regional scale [11,30]. It was found that trace metals like Pb, Cd, Cu, Zn, Cr, and Ni have accumulated in the farmland soils of Tancheng County, with average topsoil concentrations 1.62–1.77 times higher than their background concentrations in subsoil. However, they were still much lower than the limits affecting agricultural production and human health according to Chinese Environmental Quality Standard for Soils [29]. Some outliers existed; Zn concentration was found to display a larger variation coefficient than that of Ni concentration in the topsoil, which was different from the case in the subsoil. This difference partially reflected the impact by anthropogenic activities, implying that more anthropogenic Zn may have been introduced into the regional soils. In fact, soil pollution by trace metals has long been a widespread and serious problem in China [31]. It is interesting to note that the average topsoil concentrations of most trace metals in Tancheng County were much lower than those observed in megacities such as Shenyang, Wuhan, and Guangzhou (Table 1). This can be explained by the fact that long-term industrialization and urbanization have resulted in a distinct accumulation of trace metals in urban soils [32]. The relative lower levels of trace metals in some other small counties or towns further supported our explication. However, trace metals also exhibited variable concentrations among those counties or towns, even though they were analyzed on a similar spatial scale (Table 1). In general, the above comparisons reflect the complexity of trace metal sources and their impact on trace metal accumulation in soil.

Although the knowledge of total concentrations gives us an integrated view of regional soil pollution, it cannot provide any information on the quantitative discrimination of natural and anthropogenic trace metals. By calculating EF values, the anthropogenic impact on trace metal accumulation in the topsoil was further assessed quantitatively. EF calculation is constrained by some requirements, and the proper selection of reference metal is especially important [18]. In this study, Al as a reference metal was properly tested by a combination of two independent analyses, which guaranteed the reliability of the quantitative assessment (Table 2). Results showed that the EF values were varied among different trace metals, but they all had an average value greater than 1.00 (Figure 2). Evidently, these results could be interpreted by an existing anthropogenic impact. With these EF values, the anthropogenic contributions to trace metal accumulation in the topsoil were further estimated. For Pb, Cd, and Zn, the estimated average contribution values were much higher than our previous reports for a rural region in Northeast China [33]. In general, this finding well supports the conclusion that soils in industrial or urban regions tend to receive higher trace metal inputs from anthropogenic sources than do soils in rural regions [34]. However, lower anthropogenic contribution values were still obtained for Cu and Ni. A possible reason for this is that both Cu and Ni in the rural region of Northeast China have lower soil background concentrations than those in the present study area, which therefore are more sensitive to anthropogenic impact and have higher anthropogenic contribution values. According to Figure 3, the anthropogenic contribution values of Pb, Cr, and Ni were observed to display a similar spatial pattern in this region, but they were generally different from those of Cd, Cu, and Zn. The spatial distribution maps strongly implied that these trace metals may have different anthropogenic sources, which will be discussed more in the subsequent section.

### 4.2. Identification of the Major Anthropogenic Sources and Their Spatial Impacts

With the quantitative analyses, it can be found that the accumulations of Pb, Cd, Cu, Zn, Cr, and Ni in the regional soils have been impacted by anthropogenic activities, although the impacts still do not exceed their natural contributions. Based on EF values, two principal components were extracted by applying the PCA method, which indicated two important anthropogenic sources for these trace metals (Table 3). The first principal component PC1 was primarily dominated by Pb, Cr, and Ni, indicating that these three trace metals may have a common anthropogenic source. This conclusion was well verified by spatial distribution maps. Previous studies have suggested that the PCA combined with GIS mapping can be an effective tool for source identification [35]. It was found that the spatial distribution pattern of PC1 was generally characterized by high values in the central urban area, which coincided with the spatial distribution patterns of anthropogenic contributions to Pb, Cr and, Ni accumulation in the topsoil (Figure 4a). The urban area is the most densely populated and industrialized area in Tancheng Country. Industrial activities in this area emit large amounts of dust containing Pb, Cr, and Ni, and can result in soil pollution with these trace metals through subsequent deposition. In addition, emissions from transportation and coal combustion have proven to be significant contributors of atmospheric Pb deposition in urban environments [36]. Therefore, it is reasonable to infer that the anthropogenic Pb, Cr, and Ni sources were mostly associated with atmospheric deposition from the central urban area. Although the preliminary industrial system was established only in recent years, its impact on anthropogenic Pb, Cr, and Ni accumulation in the regional soils has exceeded the long-term agricultural practices.

The second principal component PC2 mainly included Cd, Cu, and Zn, which thus can be defined as an anthropogenic source, specifically related to the agricultural practices occurring in the region for a long period. Long-term agricultural practices can exacerbate the trace metal accumulation in soils due to excessive applications of fertilizers, pesticides, and manures, which contain a variety of trace metals as impurities [11]. Also, sewage irrigation is another important anthropogenic source of trace metals entering Chinese farmland soils, especially in water-limited regions [31]. However, it cannot be a main source in this region because all the irrigation water comes from natural rainfall, surface water and groundwater. By comparison, both Cu and Zn were observed to exhibit more obvious spatial variability of anthropogenic contribution values than that of Cd in the topsoil (Figure 3). This observation further revealed that the three trace metals may originate from two different agricultural sources, although they were grouped into one principal component by PCA. For Cu and Zn, they are the typical marker elements of animal manure application. The two metals are supplemented as feed additives to promote animal growth and control diseases, but approximately 90% of them are excreted by animals, resulting in metal-rich manures [37]. It was reported that animal manure application represented up to 69% and 51% of total Cu and Zn inputs into Chinese farmland soils, respectively [9]. According to our investigation, there were indeed some pig factories located in the rural northern and southeastern areas of this county. In most cases, the pig manure produced by these factories would be directly transported to nearby farmlands, where they were applied for a purpose of improving soil quality. By contrast, the application of chemical fertilizers is much more popular in this region. In particular, the application of phosphorus fertilizer has already been highlighted as a significant contributor to Cd accumulation in farmland soils [38]. Fertilization tends to be regionally similar [39], and the low spatial variability of anthropogenic Cd contribution was most likely attributed to the extensive application of chemical fertilizers in this region. As a result, fertilizer application can be identified as a major anthropogenic source, which has an important impact on Cd accumulation in the topsoil. This result was different from a national-scale study by Luo et al., who presented that manure application was the major source of anthropogenic Cd input into Chinese farmland soils [9]. Such a difference reflects the fact that trace metal sources and their impact can vary greatly at different spatial scales. In the present study, Cd generally had a higher anthropogenic contribution value than other trace metals, except for Pb that was mainly contributed by atmospheric deposition. With continuous improvement in pollution control devices, energy conservation, and the usage of clean fuels, the anthropogenic contribution to trace metals by atmospheric deposition is expected to decrease; contrarily, the trace metal inputs from phosphorus fertilizer will most likely increase due to continuous depletion of high quality phosphorus rock around the world [39].

## 5. Conclusions

The integrated application of EF, PCA, and GIS mapping is an effective approach to achieve the spatial assessment of anthropogenic impact on trace metal accumulation in regional soils. Results obtained in this study showed that the accumulations of Pb, Cd, Cu, Zn, Cr, and Ni in the farmland soils of Tancheng County have been impacted by anthropogenic activities, which could contribute up to 40.83% of total metal concentration. The anthropogenic sources of Pb, Cr, and Ni were mostly associated with atmospheric deposition from the central urban area. However, trace metals like Cd, Cu, and Zn were further confirmed to originate from different agricultural sources. For Cu and Zn, their anthropogenic inputs were mostly related to pig manure application in the rural northern and southeastern areas. The extensive application of chemical fertilizers, especially the application of phosphorus fertilizer was the major contributor to anthropogenic Cd accumulation in the region. Overall, this study provided a valuable reference for the local government, which will be helpful in designing optimal soil pollution control policies and guaranteeing sustainable agricultural development.

## Figures and Tables

**Table 1:** Descriptive statistics of trace metal concentrations in the topsoil and subsoil.

Trace Metals (mg/kg)	Parameters	Pb	Cd	Cu	Zn	Cr	Ni
Study area	Minimum	33.10	0.15	23.54	68.09	41.13	24.41
(Topsoil, *n* = 97)	Maximum	56.10	0.22	42.13	131.30	68.81	40.69
	Mean	43.34	0.19	30.51	94.74	53.23	34.75
	S.D ^a^	8.34	0.03	5.73	21.85	9.27	7.21
	V.C ^b^	19.24	15.79	18.78	23.06	17.41	20.75
Study area	Minimum	18.95	0.08	16.17	43.80	27.46	15.72
(Subsoil, *n* = 25)	Maximum	26.69	0.12	20.96	63.75	35.81	26.19
	Mean	24.46	0.11	18.83	55.59	31.62	21.33
	S.D ^a^	2.48	0.02	2.84	8.81	3.19	4.11
	V.C ^b^	10.14	18.18	15.08	15.85	10.09	19.27
A county in Northwest China [22]	Mean	24	0.20	30	83	71	32
A county in Southwest China [23]	Mean	29.41	0.38	26.55	91.20	76.48	35.79
A town in East China [24]	Mean	31.41	0.11	31.60	61.13	86.38	34.93
A town in North China [25]	Mean	28.29	0.24	35.98	93.31	100.73	38.14
Shenyang [26]	Mean	116.76	1.10	92.45	234.80	67.90	—
Wuhan [27]	Mean	301.70	3.98	60.85	86.40	152.78	52.87
Guangzhou [28]	Mean	109	0.5	63	117	—	26
Chinese environmental quality standard for soils ^c^		250	0.3	50	200	150	40

^a^ Standard deviation. ^b^ Variation coefficient (%). ^c^ Limits affecting agricultural production and human health according to Chinese Environmental Quality Standard for Soils [29].

**Table 2:** Spearman correlation coefficients of Fe, Al, and their chemical fractions in the subsoil (*n* = 25).

Metal	Spearman Correlation Coefficients						Chemical Fractions % (Mean ± S.D)			
	Pb	Cd	Cu	Zn	Cr	Ni	Acid-Soluble	Reducible	Oxidizable	Residual
Fe	0.573	0.611	0.458	0.418	0.526	0.406	6.23 ± 0.71	18.21 ± 1.35	12.36 ± 1.21	63.20 ± 5.17
Al	0.813	0.876	0.795	0.729	0.842	0.704	2.68 ± 0.28	8.13 ± 1.14	5.06 ± 0.53	84.13 ± 7.75

**Table 3:** Explanation of total variance and component matrix for trace metals in the topsoil. PC = principal component.

Initial Eigenvalues				Element	Rotated Component Matrix	
Component	Total	% of Variance	Cumulative %		PC1	PC2
	Explanation of Total Variance				Component Matrixes	
1	3.191	53.182	53.182	Pb	0.974	−0.140
2	2.364	39.402	92.584	Cd	−0.235	0.866
3	0.275	4.585	97.170	Cu	0.092	0.961
4	0.077	1.279	98.449	Zn	−0.073	0.967
5	0.061	1.024	99.472	Cr	0.975	0.060
6	0.032	0.528	100.000	Ni	0.967	−0.135

## Supplementary Materials

The following is available online at http://www.mdpi.com/1660-4601/15/9/2052/s1, Table S1: Semivariogram models for anthropogenic trace metal contribution values and principal component scores.
