# Supplementary material for: Spatial Assessment of Anthropogenic Impact on Trace Metal Accumulation in Farmland Soils from a Rapid Industrializing Region, East China
Source: Int J Environ Res Public Health. 2018 Sep 19;15(9):2052. doi: 10.3390/ijerph15092052 (PMC6163759; doi:10.3390/ijerph15092052)
Supplement: Supplementary file 1 [file ijerph-15-02052-s001.pdf]

International Journal of
Environmental Research and Public Health

MDPI

Supplementary

Spatial Assessment of Anthropogenic Impact on Trace Metal Accumulation in Farmland Soils from a Rapid Industrializing Region, East China

Wei Jiao [1,*], Yong Niu [2], Yuan Niu [2], Hengyu Hu [1] and Ruiping Li [3,*]

[1] Shandong Provincial Key Laboratory of Water and Soil Conservation and Environmental Protection, College of Resources and Environment, Linyi University, Linyi 276000, China; hhyu01@163.com
[2] Institute of Lake Environment, Chinese Research Academy of Environmental Sciences, Beijing 100012, China; ny0626@outlook.com (Y.N.); niuyuan@craes.org.cn (Y.N.)
[3] School of Geography and Tourism, Qufu Normal University, Qufu 273100, China
* Correspondence: jiaowei19856261@163.com (W.J.); liruiping858@163.com (R.L.)

Table S1. Semivariogram models for anthropogenic trace metal contribution values and principal component scores. MSE = mean standardized prediction error and RMSSE = root-mean-square standardized prediction error.

	Model	C_0	Sill	Range (km)	MSE	RMSSE
Pb	Gaussian	0.102	1.117	2.45	0.047	1.079
Cd	Exponential	0.004	0.546	2.37	-0.008	0.968
Cu	Gaussian	0.082	0.656	1.69	-0.030	1.003
Zn	Gaussian	0.113	1.061	1.51	-0.005	0.903
Cr	Spherical	0.136	1.209	2.27	0.052	0.918
Ni	Spherical	0.152	1.058	2.03	0.009	0.901
PC1	Spherical	0.141	1.454	2.61	0.016	0.932
PC2	Gaussian	0.205	0.744	1.86	-0.004	0.941

© 2018 by the authors. Submitted for possible open access publication under the terms and conditions of the Creative Commons Attribution (CC BY) license (http://creativecommons.org/licenses/by/4.0/).
